# Neutralization assay with SARS-CoV-1 and SARS-CoV-2 spike pseudotyped murine leukemia virions

**DOI:** 10.1186/s12985-020-01472-1

**Published:** 2021-01-04

**Authors:** Yue Zheng, Erin T. Larragoite, Elizabeth S. C. P. Williams, Juan Lama, Isabel Cisneros, Julio C. Delgado, Patricia Slev, Jenna Rychert, Emily A. Innis, Mayte Coiras, Matthew T. Rondina, Adam M. Spivak, Vicente Planelles

**Affiliations:** 1 Department of Pathology, University of Utah School of Medicine, Salt Lake City, UT USA; 2 RetroVirox, Inc., San Diego, CA USA; 3 Associated Regional and University Pathologists (ARUP) Laboratories, Salt Lake City, UT USA; 4 AIDS Immunopathology Unit, National Center of Microbiology (CNM), Instituto de Salud Carlos III, Madrid, Spain; 5 Department of Human Genetics, University of Utah School of Medicine, Salt Lake City, UT USA; 6 Department of Medicine, University of Utah School of Medicine, Salt Lake City, UT USA

**Keywords:** COVID-19, Coronavirus, SARS, SARS-CoV-2, Neutralization assay, Pseudotyped virus, Spike, Murine leukemia virus, Antibody

## Abstract

**Background:**

Virus neutralization by antibodies is an important prognostic factor in many viral diseases. To easily and rapidly measure titers of neutralizing antibodies in serum or plasma, we developed pseudovirion particles composed of the spike glycoprotein of SARS-CoV-2 incorporated onto murine leukemia virus capsids and a modified minimal murine leukemia virus genome encoding firefly luciferase. This assay design is intended for use in laboratories with biocontainment level 2 and therefore circumvents the need for the biocontainment level 3 that would be required for replication-competent SARS-CoV-2 virus. To validate the pseudovirion assay, we set up comparisons with other available antibody tests including those from Abbott, Euroimmun and Siemens, using archived, known samples.

**Results:**

11 out of 12 SARS-CoV-2-infected patient serum samples showed neutralizing activity against SARS-CoV-2-spike pseudotyped MLV viruses, with neutralizing titers-50 (NT_50_) that ranged from 1:25 to 1:1,417. Five historical samples from patients hospitalized for severe influenza infection in 2016 tested negative in the neutralization assay (NT_50_ < 25). Three serum samples with high neutralizing activity against SARS-CoV-2/MLV pseudoviruses showed no detectable neutralizing activity (NT_50_ < 25) against SARS-CoV-1/MLV pseudovirions. We also compared the semiquantitative Siemens SARS-CoV-2 IgG test, which measures binding of IgG to recombinantly expressed receptor binding domain of SARS-CoV-2 spike glycoprotein with the neutralization titers obtained in the pseudovirion assay and the results show high concordance between the two tests (R^2^ = 0.9344).

**Conclusions:**

SARS-CoV-2 spike/MLV pseudovirions provide a practical means of assessing neutralizing activity of antibodies in serum or plasma from infected patients under laboratory conditions consistent with biocontainment level 2. This assay offers promise also in evaluating immunogenicity of spike glycoprotein-based candidate vaccines in the near future.

## Introduction

Coronaviruses are a group of enveloped RNA viruses with a positive-sense single-stranded RNA genome ranging from 26 to 32 kilobases, which can cause respiratory tract infections. In December 2019, a novel coronavirus known as severe acute respiratory syndrome coronavirus 2 (SARS-CoV-2) was identified in China and has caused a global ongoing pandemic of coronavirus disease (COVID-19). To date, SARS-CoV-2 has spread to 188 countries (https://coronavirus.jhu.edu/). More than 45 million cases and 1,185,760 deaths have been reported globally at the time of this writing.

Enveloped viruses are known to efficiently package their core elements with heterologous envelope glycoproteins, giving rise to the so called 'pseudotypes' or 'pseudoviruses'. Many laboratories have successfully generated pseudotypes containing the core elements of HIV-1 [1] or MLV [2, 3] and the envelope glycoproteins of vesicular stomatitis virus [4], murine leukemia virus [5], Lassa fever virus, ebola virus, coronavirus spike glycoproteins, and others (reviewed in [6]).

For a pseudotype virus, viral attachment [7], entry, and importantly, antibody binding and neutralization sensitivity are dependent on the membrane glycoprotein provided [6]. Using a defective MLV vector genome encoding *firefly* luciferase, and a packaging vector encoding MLV gag/pol, we describe the production of pseudovirus particles containing the spike glycoprotein of SARS-CoV-2. As controls, we also produced similar particles containing SARS-CoV-1, VSV-G or HIV-1 LAI gp160.

## Materials and methods

### Cells

HEK293FT cells, SupT1 cells and Huh7 cells were purchased from ATCC. HEK293FT and Huh7 cells were cultured in Dulbecco’s modified Eagle’s medium (DMEM) (Gibco, US) supplemented with 10% FBS (Gibco, US) and 2 mM l-glutamine (Gibco, US) at 37 °C with 5% CO_2_. SupT1 cells were cultured in RPMI supplemented with 10% FBS and 2 mM l-glutamine. HEK293T-ACE2 cells were cultured in DMEM with 10% FBS, 2 mM l-glutamine and 200 μg/mL hygromycin B (ThermoFisher, US). HEK293T-ACE2 cells were a gift from Adam Bailey and Emma Winkler (Washington University).

### Plasmids

SV-Psi^−^-Env^−^-MLV [8], pHIV-1 LAI gp160 [9], pHCMV-VSV-G [4] and pSIVmac gp130 [10] were previously described. L-LUC-SN was constructed by inserting the *firefly* luciferase gene within the polylinker of pLXSN (Clonetech, cat# 631509). pSARS-CoV-1 was purchased from Sino Biologicals. pCAGGS expressing SARS-CoV-2 RBD was obtained from BEI Resources (cat#NR-52309). The plasmid pcDNA3.1-SARS-2-S-C9 was a generous gift from Tom Gallagher and expresses a codon-optimized SARS-CoV-2 spike open reading frame with a deletion in the 19 carboxy-terminal amino acids (an endoplasmic reticulum retention signal) and addition of the C9 peptide TETSQVAPA, recognized by antibody 1D4.

### Production of pseudotyped MLV

The plasmid SV-Psi^−^-Env^−^-MLV and L-LUC-SN were co-transfected with or without an envelope glycoprotein plasmid (pHCMV-VSV-G/pSARS-CoV-1/pSARS-CoV-2/pHIV-1 LAI gp160) into HEK293FT cells using Lipofectamine™ 3000 (ThermoFisher, US). Cell supernatants containing viruses were collected after 2 days of transfection. Viruses were filtered through a 0.45 μm filter (VWR, US) and centrifuged at 4 °C, 6500 rpm for 18 h over a 20% sucrose cushion. Viruses were resuspended in 500 μL cell culture medium and stored at − 80 °C. We measured a 25% loss in infectivity due to one cycle of freeze/thaw for the SARS-CoV-2 pseudotype, and no loss for the VSV-G pseudotype.

### Pseudovirus infection

HEK293FT, HEK293T-ACE2, and Huh7 cells were seeded in 96-well plates (ThermoFisher, US) the day before infection. SupT1 cells were added into a 96-well plate at the time of infection. 5 × 10^4^ cells were added to each well. Pseudotyped MLV viruses were added to the pre-cultured cells. Cells were cultured at 37 °C with 5% CO_2_ for 2 days. All cells in each well were lysed and luciferase was measured using ONE-Glo™ Luciferase Assay reagent (Promega, US). RLUs are per well of a 96-well plate.

### Human samples

Blood samples in this study were obtained from Associated Regional and University Pathologists, Inc. (ARUP) Laboratories. In all cases, samples were de-identified. Samples were tested for one or a combination of the following assays: Abbott Architect SARS-CoV-2 IgG, specific for nucleocapsid; Euroimmun Anti-SARS-CoV-2 ELISA (IgG), specific for the S1 domain of the spike glycoprotein; Siemens SARS-CoV-2 IgG, specific for RBD-binding IgG; PCR test specific for viral nucleic acid (ARUP Laboratories). Five pre-pandemic serum samples (Flu#1–5) were archived and were from patients hospitalized for severe flu respiratory infections in 2016.

### Neutralization assay

HEK293T-ACE2 cells were seeded in 96-well plates at 5 × 10^4^ cells per well the day prior to infection. Sera were serially diluted in a volume of 50 μL using DMEM with 10% FBS, 2 mM l-glutamine and 200 μg/mL hygromycin B as diluent, and pre-incubated with 50 μL of pseudotyped viruses at 37 °C for 1 h. For these infections, virus stocks were used at a dilution resulting in 50–200 RLU in the absence of serum. Cells were then infected with the serum/pseudovirion mixtures. Luciferase was measured 48 h post infection using ONE-Glo™ Luciferase Assay reagent. Neutralization titers NT_50_, NT_80_ and NT_90_ were calculated using Prism 8 (GraphPad, US).

## Results

To generate pseudovirion particles, three plasmids were co-transfected into HEK293FT cells. The first plasmid was the packaging construct, SV-Psi^−^-Env^−^-MLV; the second plasmid was L-LUC-SN, a minimal retroviral transfer vector encoding the *firefly* luciferase reporter gene; the third plasmid was an expression construct encoding one of the following membrane viral glycoproteins: SARS-CoV spike (hereafter referred to as SARS-CoV-1), SARS-CoV-2 spike, HIV-1 LAI gp160 and VSV-G. VSV-G pseudotyped virus is used as a positive control because of its high infectivity in most cell types. HIV-1 LAI gp160-pseudotyped virus is used as a negative control as it utilizes CD4 as a primary receptor, which is present in SupT1 cells but absent in HEK293FT.

**Fig. 1:**
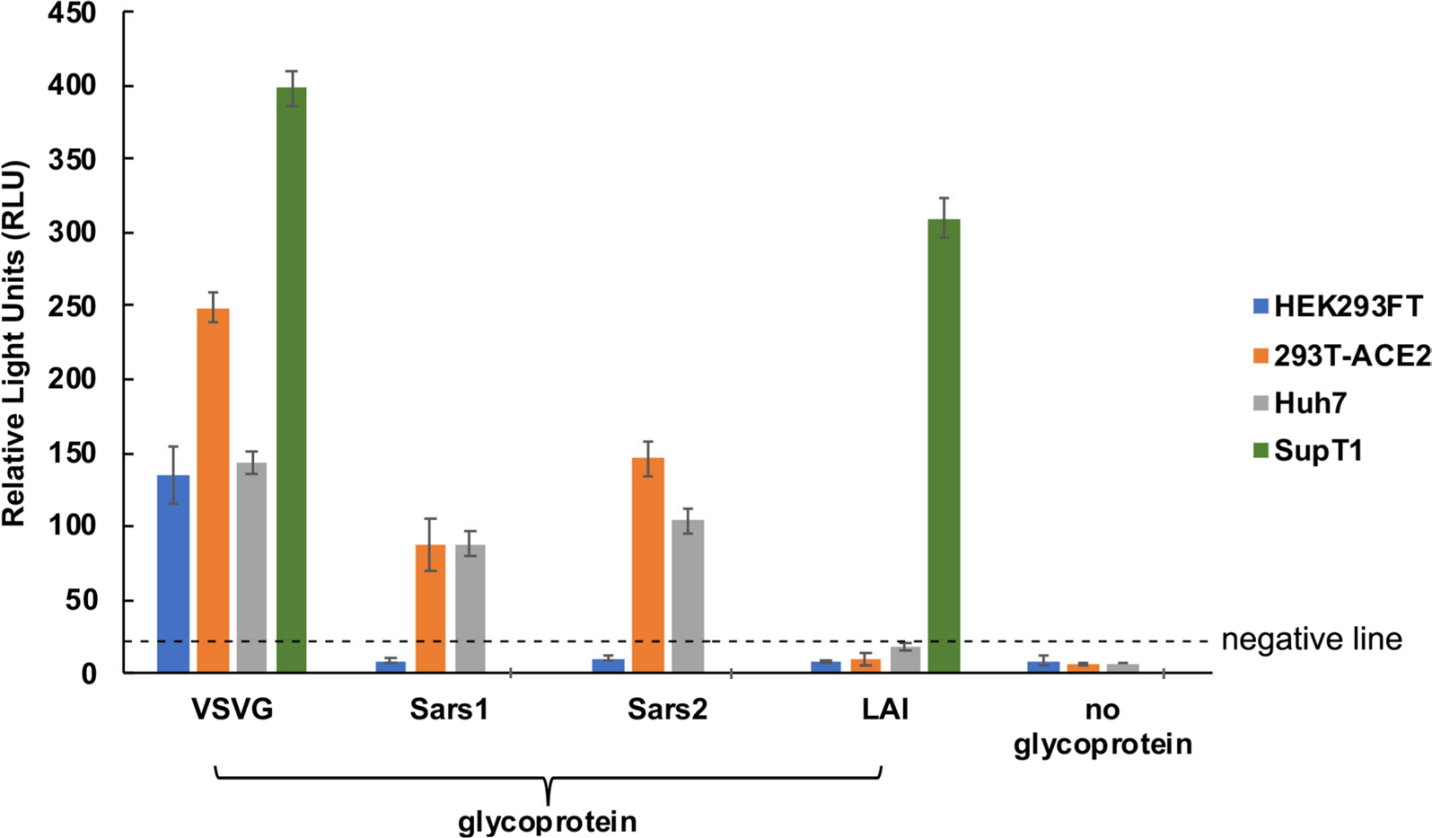
Infectivity of pseudotyped MLV Viruses. SARS-CoV-2 spike pseudotyped MLV viruses as well as VSV-G, SARS-CoV-1 spike, and HIV-1 LAIgp160 pseudotyped MLV viruses were tested on HEK293FT, HEK293T-ACE2, Huh7 and SupT1 cells. 100 μL of undiluted virus (except for VSV-G pseudotype, which was diluted 1:100) was mixed with 100 μL of medium and added to cells. Luciferase was measured at 2 days post-infection and values are per well of a 96-well plate. Negative line indicates mean + 3SD of luciferase values obtained with virions devoid of glycoprotein

Pseudotyped MLV viruses were tested on HEK293FT, HEK293T-ACE2, Huh7 and SupT1 cells. HEK293FT cells were used as a control cell line, which is known to lack susceptibility of coronavirus and HIV due to the absence of both ACE2 and CD4. As expected, VSV-G pseudotyped viruses infected all cell types and showed the highest infectivity (Fig. 1). HIV-1 LAI gp160-pseudotyped viruses only infected SupT1 cells. Both SARS-CoV-1 spike-pseudotyped and SARS-CoV-2-spike pseudotyped viruses infected HEK293T-ACE2 and Huh7 cells.

Since HEK293T-ACE2 cells showed the highest susceptibility to both SARS-CoV-1 and SARS-CoV-2 pseudotyped MLV viruses, further experiments were all performed in HEK293T-ACE2 cells. The ultimate goal of our studies was to develop a virus neutralization test based on the above pseudotyped virus. To test for neutralization activity, serum samples from 12 de-identified COVID-19 patients were tested for their ability to neutralize pseudotyped MLV viruses. Samples 1–6 were sera from patients who were tested for nucleocapsid-specific IgG (Abbott; samples 1, 8, 9 and 12), spike-specific IgG (Euroimmun; samples 2, 4, 5, 6, 8 and 9) or Nucleic Acid Amplification test (ARUP Laboratories; samples 3, and 8–11).

**Fig. 2:**
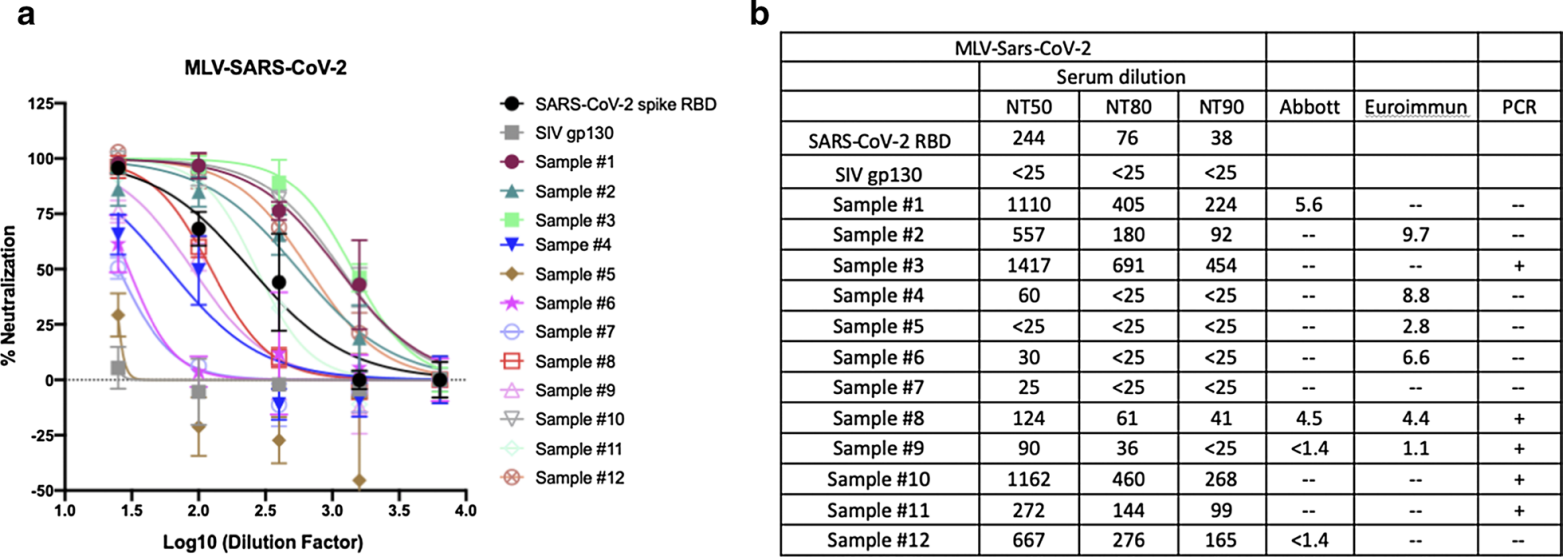
Neutralizing activity of COVID-19 patient sera against and SARS-CoV-2 pseudotyped MLV. **a** Serum samples from COVID-19 patients were pre-incubated with SARS-CoV-2 spike pseudotyped MLV at 37 °C for 1 h. Sera and virus mixture were then incubated with HEK293T-ACE2 cells for 2 days. SARS-CoV-2 spike RBD was used as a positive control. SIV gp130 was used as a negative control. Luciferase was measured to assess infection. **b** Neutralization titer 50, 80 and 90 (NT_50_, NT_80,_ NT_90_) were calculated as the reciprocal of the dilution resulting in 50, 80 and 90% neutralization, respectively. ELISA tests by Abbott and Euroimmun are not quantitative. Positive samples for the Abbott test are ≥ 1.4 and for the Euroimmun test ≥ 1.1

As a positive control and also as a standard to monitor variability between neutralization experiments, we used recombinant soluble receptor binding domain (RBD) from SARS-CoV-2 spike protein. We produced this protein via transient transfection in HEK293FT cells using a mammalian expression vector (pCAGGS) encoding amino acids 319 to 542 of from SARS-CoV-2 S1, encompassing the RBD. The apparent NT_50_ of RBD against SARS-CoV-2/MLV pseudotype was 1:244 (Fig. 2). As a negative control for neutralization, the surface glycoprotein from the simian immunodeficiency virus, SIVmac gp130 [10], was similarly produced by mammalian cell transfection.

**Fig. 3:**
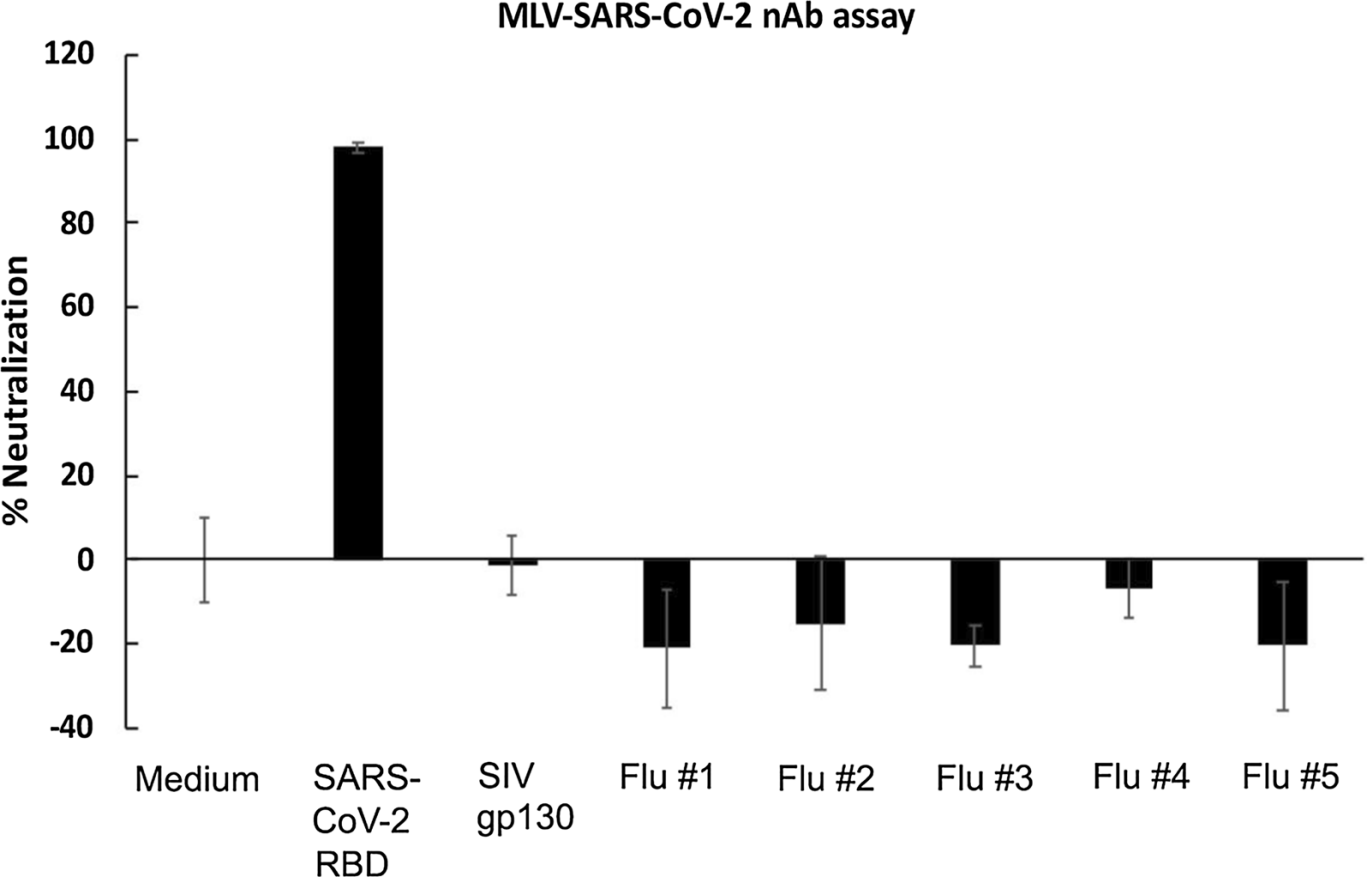
Sera from hospitalized flu patients had no neutralizing activity against SARS-CoV-2 pseudovirions. Cryopreserved serum samples from hospitalized flu patients from 2016 were pre-incubated with SARS-CoV-2 spike pseudotyped MLV at 37 °C for 1 h. Serum and virus mixture were then incubated with HEK293T-ACE2 cells for 2 days. SARS-CoV-2 RBD was used as a positive control. SIV gp130 was used as a negative control

As shown in Fig. 2, 11 out of 12 patient serum samples showed neutralizing activity against SARS-CoV-2-spike pseudotyped MLV viruses, with neutralizing titers-50 (NT_50_) that ranged from 1:25 to 1:1,417. Eight out of the 12 samples displayed detectable NT_80_ and 7 samples displayed detectable NT_90_. We also tested five historical samples from patients who were hospitalized for severe influenza infection in 2016. We tested the historical samples at the 1:25 dilution only and reported % neutralization. All of these samples tested negative in our neutralization assay (Fig. 3).

**Fig. 4:**
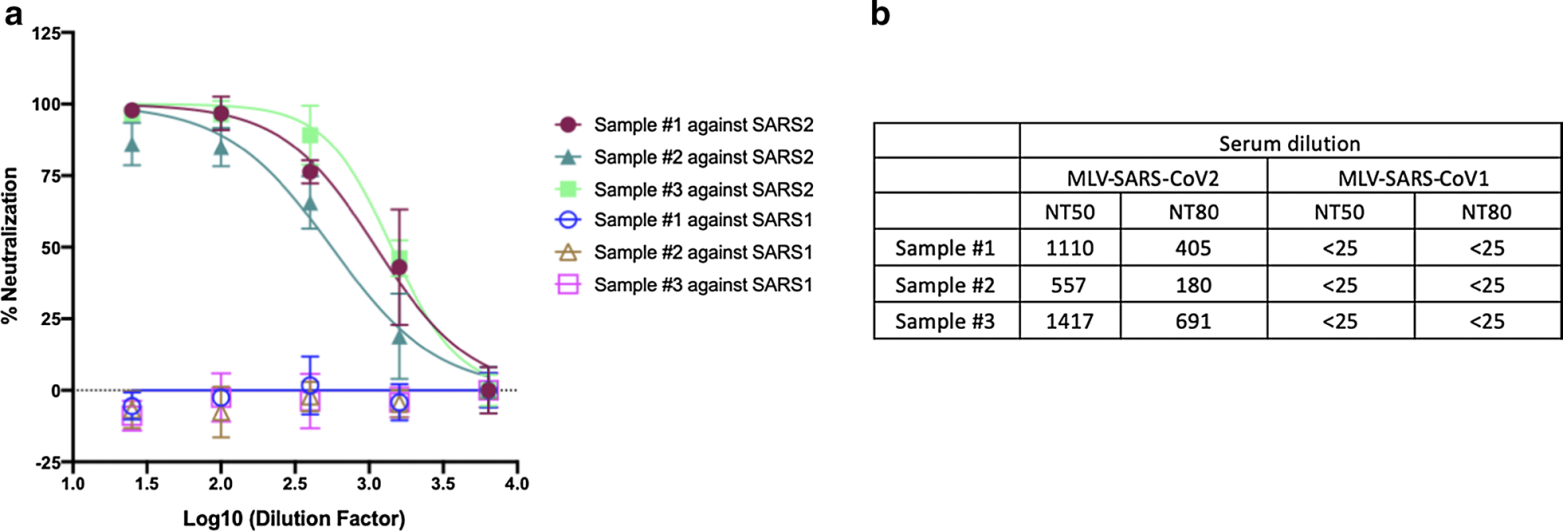
Neutralizing activity of COVID-19 patient serum against SARS-CoV-1 pseudovirions. **a** Serum from COVID-19 patients were pre-incubated with SARS-CoV-1 spike pseudotyped MLV at 37 °C for 1 h. Serum and virus mixture were then incubated with HEK293T-ACE2 cells. **b** NT_50_ and NT_80_ were calculated as above. Samples #1, 2 and 3 were tested previously (Fig. 2)

To test for specificity of neutralization, we asked whether neutralizing antibodies from SARS-CoV-2 patients would exhibit cross-reactivity against a pseudotype expressing SARS-CoV-1 (Fig. 4). We tested samples #1, 2 and 3, which had high neutralization titers. None of these sera had detectable neutralizing activity (NT_50_ < 25) against the SARS-CoV-1 pseudotype, which is consistent with previous reports [11–13].

**Fig. 5:**
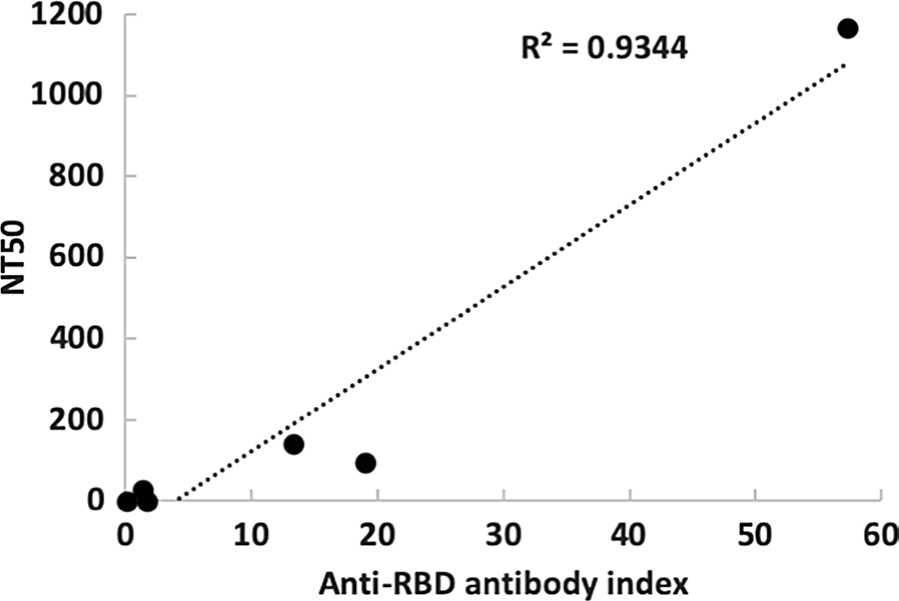
Comparison between MLV pseudotype neutralization and the Siemens SARS-CoV-2 IgG assay. Siemens SARS-CoV-2 IgG assay measuring IgG binding to recombinant receptor binding domain (horizontal axis) was compared by linear regression to the MLV pseudotype assay (vertical axis). Samples used in this experiment were #8 (shown in Fig. 2) and #13 and #15–18, not used in previous figures

It has been proposed that most neutralizing antibodies against SARS-CoV-2 act by binding the RBD of the spike glycoprotein and blocking binding to ACE-2. The Siemens SARS-CoV-2 IgG assay measures binding of IgG to recombinant RBD [14]. We wished to assess the potential correlation between our pseudovirion neutralization test and Siemens SARS-CoV-2 IgG. For this purpose, we used 6 samples and tested them in parallel in both assays. We then plotted the NT_50_ titer from the pseudovirion test against the Anti-RBD IgG index provided by the Siemens test (Fig. 5). As shown on Fig. 5, both assays can be fitted to a linear regression model with an R^2^ = 0.93.

## Discussion

The importance of neutralizing antibodies in many viral infections cannot be overstated. The presence of neutralizing antibodies due to previous infection or vaccination is in many cases expected to protect the individual from future infection and/or from severe illness. While this has been documented for many other pathogens, how neutralizing antibodies will impact SARS-CoV-2 infection and disease severity is still unclear. The availability of tests that measure antibody neutralization in the setting of SARS-CoV-2 will lead to a better understanding of the mechanisms of pathogenesis and how the immune system reacts to the presence of the virus in the organism.

Ideally, vaccines against SARS-CoV-2 will become available toward the end of 2020. For vaccines that are based on the spike glycoprotein, studies measuring antibody neutralization, together with evaluation of cellular immunity, will help to better understand and predict vaccine effectiveness. Specifically, it is imperative that we understand whether neutralizing antibodies can be correlates of protection against progression to severe disease; and, if so, whether we can establish a threshold of neutralization activity above which protection is likely. Additionally, we should be able to establish whether vaccine-induced immunity is comparable to natural immunity from infection in terms of protection from infection and disease severity.

Antibody neutralization can also be a critical factor when deciding on potential sources of convalescent immune plasma as this approach is able to provide immediate immunity in susceptible individuals [15]. In one study, a single dose of 200 mL of convalescent plasma with an antibody titer of 1:640 was transfused to 10 patients along with antiviral agents and supportive measures [16]. Symptoms in all of patients, especially fever, cough, shortness of breath, and chest pain, disappeared or largely improved one to three days after plasma infusion [16]. Being able to evaluate the titers of neutralizing antibodies in convalescent plasma sources will allow for selection of samples with highest likelihood of success.

## Conclusions

In summary, we have developed a simple and rapid assay based on pseudovirion particles, which allows for specific measurement of neutralizing titers in plasma against SARS-CoV-2 in the context of biocontainment level 2 laboratories.

## Data Availability

Plasmid constructs and methodology are available upon request. Aliquots of plasma and serum are in limiting quantities and may be available depending on amount requested.

## Abbreviations

SARS: Severe acute respiratory syndrome
SARS-CoV-2: Severe acute respiratory syndrome coronavirus 2
COVID-19: Coronavirus disease 2019
DMEM: Dulbecco’s modified Eagle’s medium
FBS: Fetal bovine serum
RBD: Receptor binding domain
NT: Neutralizing titer
MLV: Murine leukemia virus
VSV-G: Vesicular stomatitis virus glycoprotein
HIV: Human immunodeficiency virus
ACE2: Angiotensin Converting Enzyme 2
